# Supplementary material for: An automatic adaptive method to combine summary statistics in approximate Bayesian computation
Source: PLoS One. 2020 Aug 6;15(8):e0236954. doi: 10.1371/journal.pone.0236954 (PMC7410215; doi:10.1371/journal.pone.0236954)
Supplement: S1 File — (PDF) [file pone.0236954.s001.pdf]

Supplementary material: An automatic adaptive method to combine summary statistics in approximate Bayesian computation

Jonathan U. Harrison[1*], Ruth E. Baker[2]

1 Mathematical Institute, Mathematical Sciences Building,
University of Warwick, Coventry, CV4 7AL, U.K.
2 Mathematical Institute, Andrew Wiles Building,
University of Oxford, Oxford, OX2 6GG, U.K.

* jonathan.u.harrison@warwick.ac.uk

A Theoretical justification

We will require the following results:

Definition (Uniformly Lebesgue approximable function). Let $g \in L_1(E)$ for $E \subset \mathbb{R}^d$. g is *uniformly Lebesgue approximable* on E if, for any sequence $R_n \to 0$ and any $\delta > 0$, $\exists\, n = n_0(\delta) \in \mathbb{Z}^+$ (independent of x) such that if $n > n_0$, then for almost all $x \in E$,

$$g(x) - \delta < \frac{\int_{\mathcal{B}(x,R_n)\cap E} g(t)\mathrm{d}t}{\mathcal{V}(\mathcal{B}(x,R_n)\cap E)} < g(x) + \delta,$$

where $\mathcal{B}(x,R)$ is the closed ball around point $x \in \mathbb{R}^d$ with radius R, and $\mathcal{V}(\mathcal{B}(x,R))$ is the volume of the ball.

Lemma 1. *[1]*

Suppose that $k \geq 2$ and that $\mathcal{M} = supp(p)$. Assume that: (a) q is bounded above; (b) p is bounded away from zero; (c) p is uniformly Lebesgue approximable; (d) $\exists\, \delta_0$ such that $\forall \delta \in (0, \delta_0)$ $\int_{\mathcal{M}} H(x,p,\delta,1/2)p(x)\mathrm{d}x < \infty$; (e) $\int_{\mathcal{M}} ||x-y||^\gamma p(y)\mathrm{d}y < \infty$ for almost all $x \in \mathcal{M}$,

$\int\int_{\mathcal{M}^2} ||x-y||^\gamma p(y)p(x)\mathrm{d}y\mathrm{d}x < \infty$, where

$H(x,p,\delta,\psi) = \sum_{j=0}^{k-1} \left(\frac{1}{j!}\right)^\psi \Gamma(\frac{1}{2}+j\psi) \left(\frac{p(x)+\delta}{p(x)-\delta}\right)^{j\psi} (p(x)-\delta)^{-(\frac{1}{2})} \left((1-\delta)\psi\right)^{-(\frac{1}{2})-j\psi}$.

Then

$$\lim_{n\to\infty} \mathbb{E}\left[\left(\hat{D}_h\left(X_{1:N}||Y_{1:N}\right) - D_h\left(p||q\right)\right)^2\right] = 0. \tag{1}$$

The proof of this results relies on constructing an integrable function as a bound such that Lebesgue's dominated convergence theorem can be applied. See Póczos and Schneider [1] for details. Lemma 3 specifies L_2 consistency of the nearest neighbour estimator, which ensures that the estimates of the distance between p and q become more concentrated around the true values as more samples are used.

Theorem 2. *Assume E is finite, $|E| = \chi$. Assume the conditions of Lemma 3 hold for distributions p and $q^{(\mathbf{w})}$. Assume that a unique $\mathbf{w}^*$ maximizes $D_h\left(p||q^{(\mathbf{w})}\right)$ and arrange parameter values $\mathbf{w}^j$ for $j \in \{1,\dots,\chi\}$ in order such that they are descending in $D_h\left(p||q^{(\mathbf{w})}\right)$. That is $\mathbf{w}^1 = \mathbf{w}^*$, using $\mathbf{w}^2$ gives the next biggest value and so on. Then*

$$\lim_{n\to\infty} \mathbb{P}\left(\operatorname*{argmax}_{\mathbf{w}\in S} \hat{D}_h\left(X_{1:N}||Y_{1:N}^{(\mathbf{w})}\right) = \mathbf{w}^*\right) = 1. \tag{2}$$

Proof. Let $\epsilon > 0$. Take $\delta < D_h\left(p||q^{(\mathbf{w}^*)}\right) - D_h\left(p||q^{(\mathbf{w}^2)}\right)$. Using Lemma 3, we have L_2 convergence for the estimator $\hat{D}_h$ and, which therefore implies convergence in probability. Therefore $\exists M \in \mathbb{N}$ such that $\forall n \geq M$

$$\mathbb{P}\left(\left|\hat{D}_h\left(X_{1:N}||Y_{1:N}^{(\mathbf{w})}\right) - D_h\left(p||q^{(\mathbf{w})}\right)\right| > \delta\right) < \epsilon.$$

Therefore $\forall N \geq M$

$$\mathbb{P}\left(\operatorname*{argmax}_{\mathbf{w}\in E} \hat{D}_h\left(X_{1:N}||Y_{1:N}^{(\mathbf{w})}\right) = \mathbf{w}^*\right) > 1 - \epsilon.$$

□

If we make the (generally unrealistic) assumption that the space of possible parameters, E, is finite, then we are able to show that, in the limit of a large number of samples, we can recover the optimum parameters with probability 1. We next explore how this can be extended to a compact, continuous space of parameters, E, provided we make assumptions about the structure of dependence of the distance estimator on parameters $\mathbf{w} \in E$. We show that assumptions about this dependence structure of the estimator on the parameters can be justified by considering some of the details of Algorithm 2.

Lemma 3. *Consider the estimator of the Hellinger distance as a function of parameters,* $\mathbf{w}$*, such that*

$$L(\mathbf{w}) = \hat{D}_h \left(X_{1:N} || Y_{1:N}^{(\mathbf{w})} \right).$$

Then $L(\mathbf{w})$ *is piecewise constant with respect to parameters* $\mathbf{w} \in E$*, with finitely many discontinuities.*

Proof. Suppose that, according to Algorithm 2, at generation t, we have generated pseudo data $\{\mathbf{x}^{i*}\}_{i=1}^{N}$, which we summarize via summary statistics $s(\mathbf{x}^{i*}) = (s_1, \ldots, s_\kappa) \in \mathbb{R}^\kappa$ and κ is the number of summary statistics used to summarise the model output. The parameters $\mathbf{w} \in \mathbb{R}^\kappa$ are summary statistic weights, and these are used within a weighted Euclidean distance function

$$\begin{aligned} d_{\mathbf{w}}\left(s(\mathbf{x}), s(\mathbf{y})\right) &= \sum_{j=1}^{\kappa} w_i^2 (s_j - s_j(\mathbf{y}))^2 \\ &= (s(\mathbf{x}) - s(\mathbf{y}))^\top \Sigma_w^\top \Sigma_w (s(\mathbf{x}) - s(\mathbf{y})) \\ &= \left(\Sigma_w s(\mathbf{x}) - \Sigma_w s(\mathbf{y})\right)^\top \left(\Sigma_w s(\mathbf{x}) - \Sigma_w s(\mathbf{y})\right), \end{aligned}$$

where $\Sigma_w = \text{diag}(\mathbf{w})$, to compare the pseudo data with observed data. We note that this is equivalent to stretching the space in which the pseudo data lies via the matrix Σ_w, and using the usual Euclidean distance.

Consider a small perturbation in parameter space $\mathbf{w} = \mathbf{w}_0 + \epsilon$, with $||\epsilon|| \ll 1$. Then

$$\Sigma_{\mathbf{w}} = \text{diag}(\mathbf{w}_0 + \epsilon) = \text{diag}(\mathbf{w}_0) + \text{diag}(\epsilon) = \Sigma_{\mathbf{w}_0} + \Sigma_\epsilon.$$

Using this decomposition of $\Sigma_{\mathbf{w}}$ gives, for the weighted Euclidean distance,

$$\begin{aligned} d_{\mathbf{w}_0+\epsilon}(s(\mathbf{x}), s(\mathbf{y})) &= \left(\left(\Sigma_{\mathbf{w}_0} + \Sigma_\epsilon\right) s(\mathbf{x}) - \left(\Sigma_{\mathbf{w}_0} + \Sigma_\epsilon\right) s(\mathbf{y})\right)^\top \\ &\quad \left(\left(\Sigma_{\mathbf{w}_0} + \Sigma_\epsilon\right) s(\mathbf{x}) - \left(\Sigma_{\mathbf{w}_0} + \Sigma_\epsilon\right) s(\mathbf{y})\right) \\ &= \left(\Sigma_{\mathbf{w}_0} s(\mathbf{x}) - \Sigma_{\mathbf{w}_0} s(\mathbf{y})\right)^\top \left(\Sigma_{\mathbf{w}_0} s(\mathbf{x}) - \Sigma_{\mathbf{w}_0} s(\mathbf{y})\right) \\ &\quad + \left(\Sigma_\epsilon s(\mathbf{x}) - \Sigma_\epsilon s(\mathbf{y})\right)^\top \left(\Sigma_{\mathbf{w}_0} s(\mathbf{x}) - \Sigma_{\mathbf{w}_0} s(\mathbf{y})\right) \\ &\quad + \left(\Sigma_{\mathbf{w}_0} s(\mathbf{x}) - \Sigma_{\mathbf{w}_0} s(\mathbf{y})\right)^\top \left(\Sigma_\epsilon s(\mathbf{x}) - \Sigma_\epsilon y\right) + \mathcal{O}(\epsilon^2) \\ &= d_{\mathbf{w}_0}(s(\mathbf{x}), s(\mathbf{y})) + A + A^\top + \mathcal{O}(\epsilon^2), \end{aligned}$$

where $A = \left(\Sigma_{\mathbf{w}_0} s(\mathbf{x}) - \Sigma_{\mathbf{w}_0} s(\mathbf{y})\right)^\top \left(\Sigma_\epsilon s(\mathbf{x}) - \Sigma_\epsilon s(\mathbf{y})\right)$ which is linear in ϵ. Suppose we order the pseudo-data such that $\mathbf{x}^{j*}$ is the jth closest point to the observed data based on $d_{\mathbf{w}_0}$. Provided that

$$d_{\mathbf{w}_0}(s(\mathbf{x}^{n*}), s(\mathbf{y})) - d_{\mathbf{w}_0}(s(\mathbf{x}^{(n+1)*}), s(\mathbf{y})) > A + A^\top + \mathcal{O}(\epsilon^2), \tag{3}$$

then making this perturbation in $\mathbf{w}$ will not change which parameter samples are selected, as the same pseudo data will remain closest to the observed data, $\mathbf{y}$. If the

same parameter samples are selected, then the value of $L(\mathbf{w})$ will remain constant as a function of $\mathbf{w}$ under the perturbation $\mathbf{w} = \mathbf{w}_0 + \epsilon$.

In cases where eq. (3) does not hold, there will be a jump discontinuity in $L(\mathbf{w})$ as different parameter samples are selected. This will occur finitely many times corresponding to the finite number, N, of points in the sample of pseudo data, $\{\mathbf{x}^{i*}\}_{i=1}^{N}$. □

We confirm computationally that $L(\mathbf{w})$ is piecewise constant for the test problem described in Section 4.1, and show this in Figure S1.

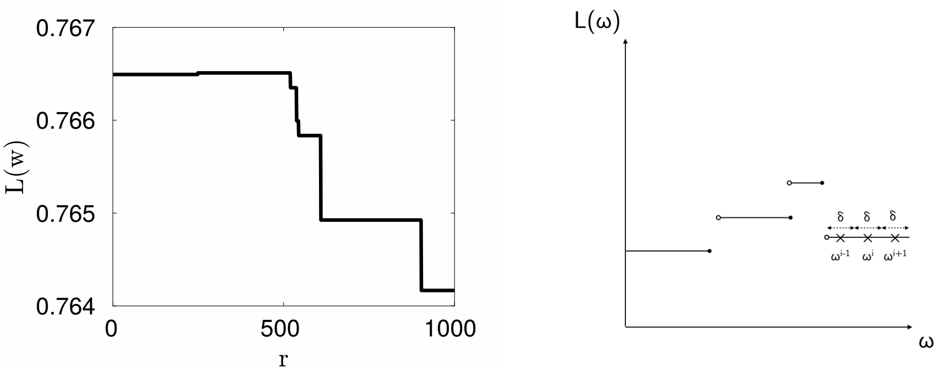

(a) (b)

Fig S1. The estimator $L(\mathbf{w}) = \hat{D}_h\left(X_{1:N}||Y_{1:N}^{(\mathbf{w})}\right)$ is piecewise constant for the toy model described in Section 4.1 when $X_{1:N}$ are samples from the prior and $Y_{1:N}^{(\mathbf{w})}$ are samples from the approximate posterior generated via ABC with summary statistics weights $\mathbf{w}$. After a single generation of ABC-SMC, we optimize $L(\mathbf{w})$ as a function of $\mathbf{w}$ to find a local maximum $\mathbf{w}^*$. In (a), we then consider the value of $L(\mathbf{w})$ on a line of parameters in parameter space $\mathbf{w} = \mathbf{w}^* + 10^{-4} r\vec{\eta}$, where $\vec{\eta} \sim \mathcal{N}(\mathbf{0}, I_\kappa)$ is a random choice of direction, I_κ is the $\kappa \times \kappa$ identity matrix and r parameterizes a line in this direction. $L(\mathbf{w})$ is piecewise constant in $\mathbf{w}$ as shown in Lemma 3. For a piecewise constant function $L(\mathbf{w})$, we can choose δ and $\mathbf{w}^j$ for $j \in \{1, \ldots, \kappa\}$ such that $L(\mathbf{w})$ is locally constant. We illustrate such a choice of δ and $\mathbf{w}^j$ in (b).

Lemma 4. *Assume $E \subset \mathbb{R}^\kappa$ is compact and that a unique $\mathbf{w}^* \in E$ maximizes $D_h\left(p||q^{(\mathbf{w})}\right)$. Assume the conditions of Lemma 3 hold for distributions p and $q^{(\mathbf{w})}$. Suppose $L(\mathbf{w})$ is piecewise constant in $\mathbf{w}$ with finitely many jump discontinuities. Then*

$$\lim_{n\to\infty} \mathbb{P}\left(\operatorname*{argmax}_{\mathbf{w}\in E} \hat{D}_h\left(X_{1:n}||Y_{1:n}^{(\mathbf{w})}\right) = \mathbf{w}^*\right) = 1.$$

Proof. We can choose a $\delta > 0$ and finitely many $\mathbf{w}^j$, $j \in \{1, \ldots, \chi\}$ such that every point $\mathbf{w}$ is within a ball of radius δ from some $\mathbf{w}^j$ (see Figure S1 and note that L is locally constant). Then $\forall \mathbf{w} \in E\ ||\mathbf{w} - \mathbf{w}^j|| < \delta \implies L(\mathbf{w}) = L(\mathbf{w}^j) = L_j$. Since there are finitely many values $\mathbf{w}^j$ corresponding to distinct unique values L^j, we can apply the result from Theorem 5 to give the required result. □

Theorem 5. *Suppose that $k \geq 2$ and that $\mathcal{M} = supp(p)$. Assume that:*
(a) q is bounded above;
(b) p is bounded away from zero;
(c) p is uniformly Lebesgue approximable;

(d) $\exists\, \delta_0$ *such that* $\forall \delta \in (0, \delta_0)$

$$\int_{\mathcal{M}} H(x, p, \delta, 1/2) p(x) \mathrm{d}x < \infty;$$

(e) $\int_{\mathcal{M}} ||x - y||^{\gamma} p(y) \mathrm{d}y < \infty$ *for almost all* $x \in \mathcal{M}$,
$\int\int_{\mathcal{M}^2} ||x - y||^{\gamma} p(y) p(x) \mathrm{d}y \mathrm{d}x < \infty$*;*
(f) $E \subset \mathbb{R}^{\kappa}$ *is compact;*
(g) a unique $\mathbf{w}^* \in E$ *maximizes* $D_h \left(p || q^{(\mathbf{w})}\right)$*.*
Then

$$\lim_{n \to \infty} \mathbb{P}\left(\operatorname*{argmax}_{w \in E} \hat{D}_h\left(X_{1:N} || Y_{1:N}^{(\mathbf{w})}\right) = \mathbf{w}^*\right) = 1. \tag{4}$$

Proof. Apply the Lemma 4 to show that $L(\mathbf{w})$ is piecewise constant in $\mathbf{w}$. Apply Lemma 5 to give the desired result. □

Theorem 6. *[2]*
Suppose that the following conditions hold:
(a) $\theta \in \mathbb{R}^{\lambda}$, $s(\mathbf{x}) \subset \mathbb{R}^{\kappa}$ *and these random variables have joint density* $\pi(\theta, s(\mathbf{x}))$ *with respect to the Lebesgue measure;*
(b) the sets $A_t = \{s(\mathbf{x}) \,|\, d_{\mathbf{w}_t^*}(s(\mathbf{x}), s(\mathbf{y})) < \epsilon_t\}$ *are Lebesgue measureable, where* w^* *are the optimal weights at generation* t*, and* ϵ_t *are the tolerances at each generation;*
(c) $\pi(s(\mathbf{y})) > 0$*;*
(d) $\lim_{t\to\infty} m(A_t) = 0$*, where* $m(\cdot)$ *represents Lebesgue measure;*
(e) the sets A_t *have bounded eccentricity. That is for any* A_t*,* $\exists$ *a ball* $B_t = \{s(\mathbf{x}) \,|\, ||s(\mathbf{x} - s(\mathbf{y})||_2 \le r_t\}$ *about* $s(\mathbf{y})$ *with radius* r_t *such that* $A_t \subset B_t$ *and* $m(A_t) \ge c\, m(B_t)$*, where* $|| \cdot ||$ *is the Euclidean or* L_2 *norm and* $c > 0$ *is a constant. (This property implies that* A_t *is contained within a ball* B_t*, but that its measure is comparable to the measure of the ball [4]).*
Then the posterior approximations converge:

$$\lim_{t\to\infty} p_{ABC,t}(\theta | s(\mathbf{y})) = p(\theta | s(\mathbf{y})) \text{ for almost all } (\theta, s(\mathbf{y})),$$

where $p_{ABC,t}(\theta|s(\mathbf{y}))$ *is the ABC posterior defined as*

$$p_{ABC,t}(\theta|s(\mathbf{y})) \propto \int p(s|\theta)\pi(\theta) \mathbb{1}[d_{\mathbf{w}_t^*}(s(\mathbf{x}), s(\mathbf{y})) \le \epsilon_t] \, \mathrm{d}\mathbf{x}.$$

Proof.

$$\begin{aligned}
\lim_{t\to\infty} p_{\mathrm{ABC},t}(\theta|s(\mathbf{y})) &= \lim_{t\to\infty} \frac{\int p(\theta, s) \mathbb{1}[s \in A_t] \, \mathrm{d}s}{\int p(\theta, s) \mathbb{1}[s \in A_t] \, \mathrm{d}s \mathrm{d}\theta} \\
&= \lim_{t\to\infty} \frac{\int_{s \in A_t} p(\theta, s) \, \mathrm{d}s}{\int_{s \in A_t} p(\theta, s) \, \mathrm{d}s \mathrm{d}\theta} \\
&= \frac{\lim_{t\to\infty} \frac{1}{m(A_t)} \int_{s \in A_t} p(\theta, s) \, \mathrm{d}s}{\lim_{t\to\infty} \frac{1}{m(A_t)} \int_{s \in A_t} p(s) \, \mathrm{d}s} \\
&= \frac{p(\theta, s(\mathbf{y}))}{p(s(\mathbf{y}))} \text{ almost everywhere} \\
&= p(\theta|s(\mathbf{y})).
\end{aligned}$$

The fourth equality above is due to the Lebesgue differentiation theorem [4], which requires bounded eccentricity and $\lim_{t\to\infty} m(A_t) = 0$. □

B Further examples

We provide here further examples to demonstrate behaviour of the adaptive ABC-SMC method presented in Algorithm 2.

B.1 Gaussian toy model

Suppose we aim to infer a single parameter, θ. We observe two summary statistics $s(\mathbf{x}) = (x_1, x_2)$, where $x_1 \sim \mathcal{N}(\theta, 0.1^2)$ and $x_2 \sim \mathcal{N}(0, 1^2)$ such that the first summary statistic is informative and the second is uninformative. We take a broad prior distribution of $\mathcal{N}(0, 100^2)$ for θ. The effect of this is that by choosing weights for summary statistics based on the prior predictive distribution, the weight for the informative summary statistic, x_1, is smaller than for the uninformative statistic, x_2. This toy model was used previously by Prangle [2] to demonstrate their weight adaption method, by comparison with fixed weights based on summary statistics simulated from the prior predictive distribution. The method from Algorithm 2 improves on this method, as shown in Figure S2, by reaching faster adaption to the scales of the summary statistics, combined with how informative they are. In addition, in Figure S2 we show the scaling of the MSE as the number of simulations increases. Here there are two summary statistics on similar scales, so weighting these equally is a sensible choice. The adaptive method of Algorithm 2 matches the scaling of MSE of the uniform choice of weights, improving upon the scaled method.

B.2 Death process

For our first test problem, we consider estimating the rate parameter for a single, first order degradation reaction:

$$\mathrm{A} \xrightarrow{k_1} \emptyset. \tag{5}$$

We will consider for this, and subsequent, test problems that time has been non-dimensionalized. Initially, we assume there are $A(0) = 10$ particles in the system, which is observed over a (non-dimensional) time period $[0, 20]$. We assume it is possible to measure the state of the system (in this case the number of molecules of species A) without observation noise at given time points $t_0, t_1, \ldots, t_n$. For this test problem, we assume that we measure at n equally spaced time intervals, where $n = 8$.

As our summary statistics, we take $s(\mathbf{x}) = [A(t_0), A(t_1), \ldots, A(t_n), z]$ where z is an observation of a random variable $Z \sim \mathcal{N}(0, \sigma^2)$ that is uncorrelated with the death process. We suppose that the scale of the variance, σ, is different to the scale of the observations of the exponential decay process, giving a simple system with a two-dimensional parameter to infer: $\theta = (k_1, \sigma)$. Note that the scale of z is determined by the standard deviation, σ, but the scale of the death process is affected by the initial condition, $A(0) = 10$, resulting in two distinct scales in these summary statistics.

Results of parameter inference for this system using ABC-SMC are shown in Figure S3, where the true parameters used are $\theta = (0.1, 0.01)$ and a prior uniform on the logarithm of each of the parameters over the interval $[10^{-3}, 10^3]$ was used. We observe similar performance in identification of the decay parameter k_1 using uniform weights, scaled weights and the adaptive choice of weights. Scaling the summary statistics with the MAD results in problems here since some summary statistics may not vary much upon simulation from the prior predictive distribution initially, resulting in $\sigma_i = 0$ for the corresponding summary statistic. To solve this, we set the weight for a given summary statistic to a large fixed value if the MAD for that summary statistic was below a small threshold value. Note that only one summary statistic provides

**Test problem**	κ	$\mathbf{A(0)}$	T	θ^*	N	α	**Repeats**	**Prior interval**
Uniform toy model	10	-	-	10	2000	0.5	1	$\log_{10}\theta \sim U[-4,4]$
Bimodal toy model	2	-	-	$(\pi/4, 5\pi/4)$	2000	0.5	1	$\theta \sim U[0,2\pi]$
Gaussian toy model	2	-	-	(0,0)	2000	0.5	1	$\theta \sim N(0,100)$
Death process	9	10	20	$(0.1, 0.01)$	2000	0.5	1	$\log_{10}\theta \sim U[-4,4]$
Dimerization	24	$(10^5, 0, 0)$	100	$(1, 0.04, 0.002, 0.5)$	2000	0.5	1	$\log_{10}\theta \sim U([-2,2]\times[-3,1] \times[-5,-1]\times[-3,1])$
Diffusion	64	$10 * \mathbb{1}_{x<0}$	20	0.1	2000	0.5	1	$\log_{10}\theta \sim U[-4,0]$

Table S1. Summary of hyperparameters used in simulations.

information about the random variable Z, whereas the other $n+1$ summary statistics (which are observations of the decay process at time points $\{t_i\}_{i=0}^{n}$) provide information about the decay of species A. The summary statistic weights chosen via the search process outlined in Algorithm 2 give rise to a posterior that outperforms the posteriors generated using uniform weights and scaled weights for the second parameter σ, since only a single summary statistic provides relevant information for this parameter.

C Robustness to a diffuse prior

We demonstrate robustness the our method to changes in the variance of the prior distribution, including the case of a very diffuse prior distribution. We use a version of the Gaussian test problem, with a prior $N(0, 10^j)$ for $j = 0, 1, 2, 3$. We have two summary statistics: an informative statistic, $N(\theta, 0.1^2)$; and an uninformative statistic, $N(0, 100^2)$. Even as the variance of the prior distribution increases, the adaptive method of Algorithm 2 remains robust, and provides a closer approximation to the posterior than the uniform weights, which are hindered by the uninformative summary statistic.

D Tolerance schedule

In Algorithm 2, we make use of the quantile α of the distribution of distances from the ABC distance function to help determine the schedule of tolerances, ϵ_t. We consider here the effect of using a fixed tolerance scheme determined in advance, versus the adaptive tolerance scheme based on the quantiles, α. Results are shown in Figure S5 for the uniform toy model of Section 4.1 indicating that Algorithm 2 is robust to different choices of tolerance scheme, including a fixed tolerance scheme determined in advance. However, we recommend in practice using a tolerance determined as a quantile α of the distance distribution, as this avoids the need to set a fixed tolerance schedule in advance when the scale of typical distances between observed and simulated data is not known in advance. A fixed tolerance scheme prevents easy comparison between uniform, scaled and adaptive weighting methods for the weights of the ABC distance function, as different numbers of model simulations are required in each case, depending on the tolerance schedule. By using the quantile α of the distribution of distances, we ensure that an equal number of model simulations are used in each case. Methods to design tolerance schedules have been investigated by Silk et al. [3] based on a threshold-acceptance rate curve and could be considered as an alternative.

References

1. B Póczos and J Schneider. On the estimation of alpha-divergences. In *Proceedings of the Fourteenth International Conference on Artificial Intelligence and Statistics*, pages 609–617, 2011.

2. D Prangle. Adapting the ABC distance function. *Bayesian Analysis*, 12(1): 289–309, 2017.

3. D Silk, S Filippi, and M P H Stumpf. Optimizing threshold-schedules for sequential approximate bayesian computation: applications to molecular systems. *Statistical Applications in Genetics and Molecular Biology*, 12(5):603–618, 2013.

4. E M Stein and R Shakarchi. *Real analysis: measure theory, integration, and Hilbert spaces.* Princeton University Press, 2009.

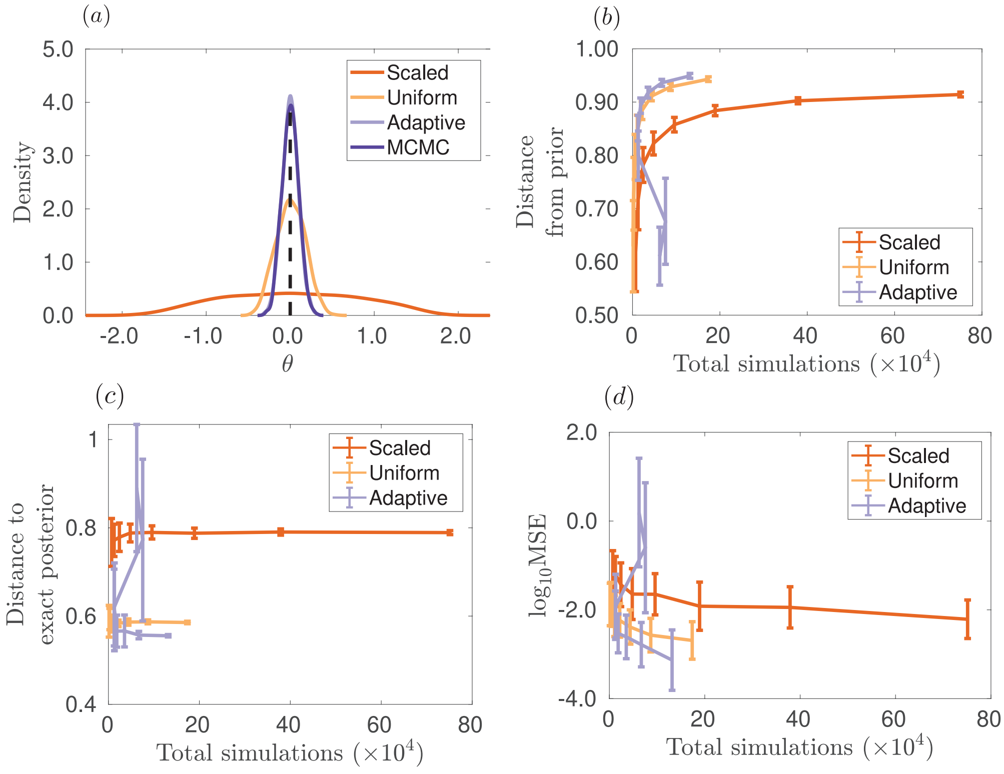

Fig S2. Posterior for parameters θ of the Gaussian toy model for different weights in the ABC distance function. ABC-SMC was used to provide estimates of the posterior, with five generations and $N = 2,000$ simulations at each generation with the posterior constructed from the closest 50% of the simulations ($\alpha = 0.5$). Metrics to evaluate the performance of Algorithm 2 are shown in (b), (c), and (d) as N varies resulting in different total numbers of simulations from the model. Results are averaged over 40 repeated runs. In (b), is shown the Hellinger distance from the prior to approximate posterior distribution, which is maximized directly within Algorithm 2. In (c), the Hellinger distance from the approximate posterior to the exact posterior distribution (as sampled via MCMC) is shown. A lower value of this distance indicates a better approximation to the posterior. In (d), the MSE from the approximate posterior mean to the exact posterior mean is shown. The orange, yellow and light purple lines show the scaled, uniform and adaptive methods, respectively. For comparison as a gold standard for this problem, the dark purple line shows the posterior obtained with MCMC using the exact likelihood without any ABC approximation. The true parameter value used to simulate the observed data is indicated by the vertical dashed black line.

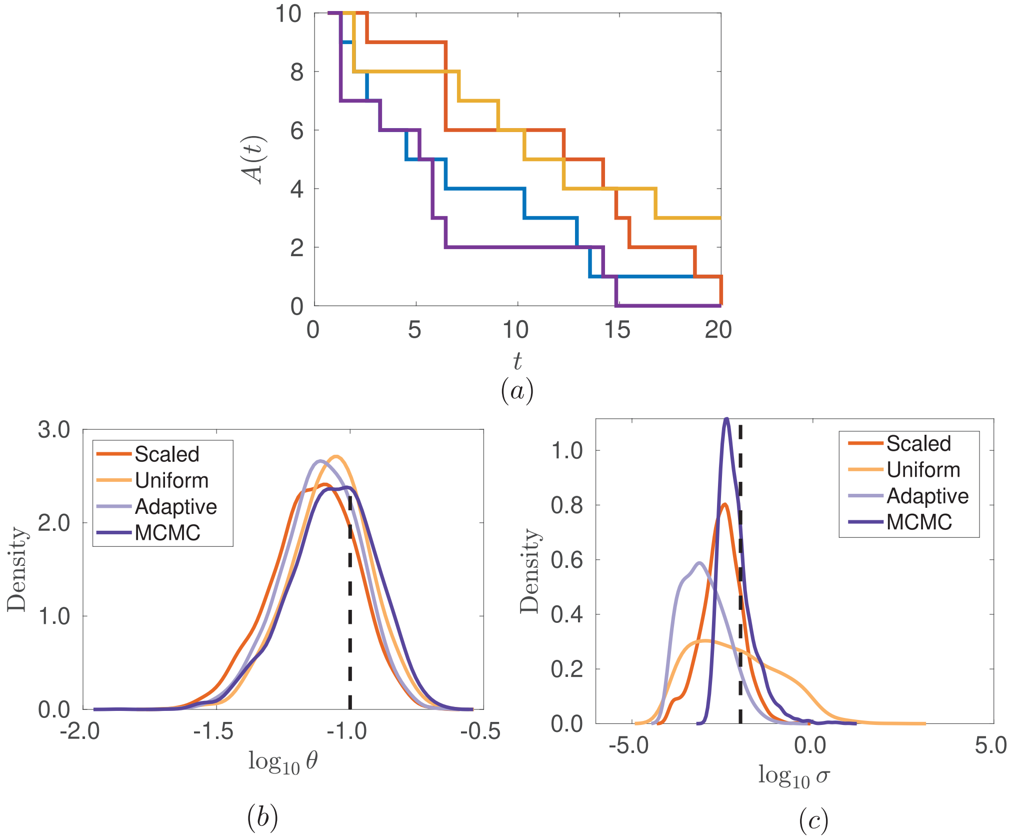

Fig S3. Posteriors for parameters k_1 and σ in the death process test problem for different weights in the ABC distance function. ABC-SMC was used to provide estimates of the posterior, with five generations and $N = 2000$ simulations at each generation with the posterior constructed from the closest 50% of the simulations ($\alpha = 0.5$). (a) shows typical output from the model for the true parameters. The posteriors for k_1 are given in (b) and for σ in (c). The orange, yellow and light purple lines show the scaled, uniform and adaptive method,s respectively. For comparison as a gold standard for this problem, the dark purple line shows the posterior obtained with MCMC using the exact likelihood without any ABC approximation. The true parameter value used to simulate the observed data is indicated by the vertical dashed black line.

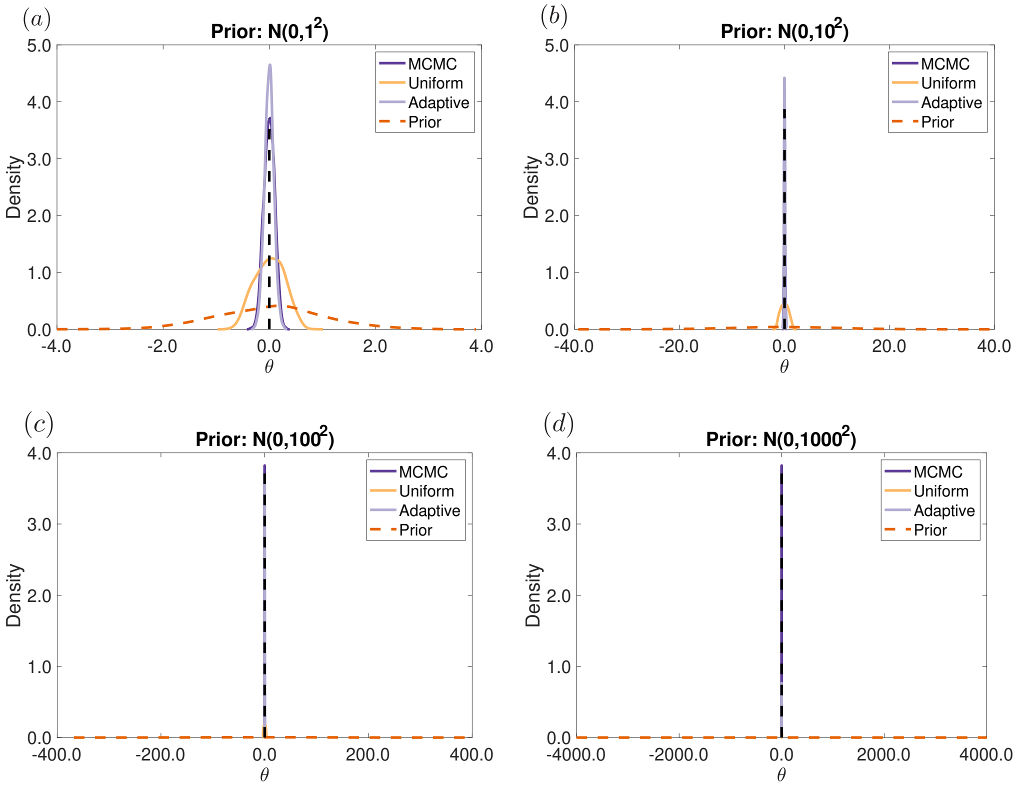

Fig S4. Increasing the variance of the prior distribution for a version of the Gaussian test problem shows the method of Algorithm 2 is robust to a broadly spread prior distribution. A prior $N(0, 10^j)$ for $j = 0, 1, 2, 3$ is assumed. Two summary statistics are used: an informative statistic, $N(\theta, 0.1^2)$; and an uninformative statistic, $N(0, 100^2)$. Results shown are approximate posterior distributions after 10 generations of ABC-SMC. The yellow and light purple lines show the uniform and adaptive methods, respectively. For comparison as a gold standard for this problem, the dark purple line shows the posterior obtained with MCMC using the exact likelihood without any ABC approximation. The true parameter value used to simulate the observed data is indicated by the vertical dashed black line. In all plots, the light purple and dark purple lines are overlapping showing the adaptive method provides a close approximation to the exact posterior distribution.

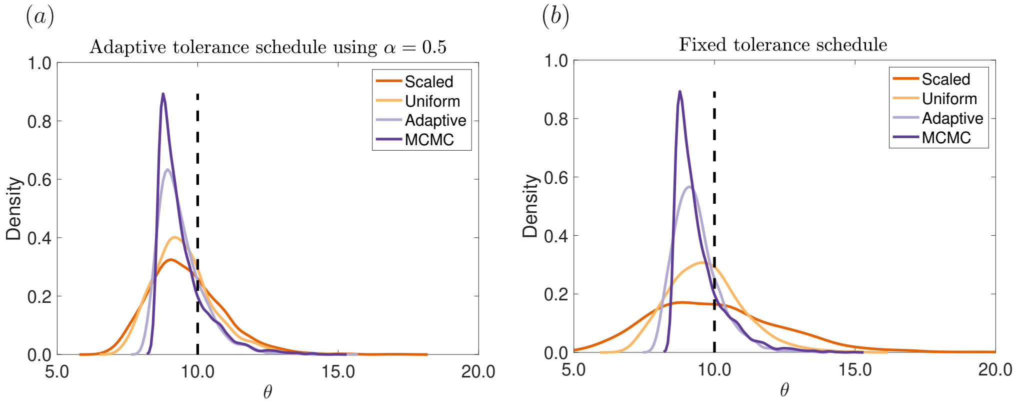

Fig S5. Comparison of an adaptive tolerance schedule (a) based on a quantile of the distribution of distances from the ABC distance function, and a fixed tolerance schedule (b). Similar results are obtained for the posterior distributions in each case indicating robustness to different tolerance schedules. The uniform toy model of Section 4.1 is used with parameters as in Figure 1 and the same dataset for both (a) and (b). The tolerance schedule used is $[\infty, 10^8, 5 \times 10^7, 2 \times 10^7, 10^7]$ for the adaptive method of Algorithm 2 over five generations. For other weighting methods, the schedule $[\infty, 100, 50, 20, 10]$ is used instead. Direct comparison between methods based on these results will not offer a fair comparison as the number of simulations drawn will vary between methods. The orange, yellow and light purple lines show the scaled, uniform and adaptive methods, respectively. For comparison as a gold standard for this problem, the dark purple line shows the posterior obtained with MCMC using the exact likelihood without any ABC approximation. The true parameter value used to simulate the observed data is indicated by the vertical dashed black line.
