# Supplementary material for: An automatic adaptive method to combine summary statistics in approximate Bayesian computation
Source: PLoS One. 2020 Aug 6;15(8):e0236954. doi: 10.1371/journal.pone.0236954 (PMC7410215; doi:10.1371/journal.pone.0236954)
Supplement: Attachment — Submitted filename: ref-PONE-D-20-06109_reviewer.pdf [file pone.0236954.s002.pdf]

An automatic adaptive method to combine summary statistics in approximate Bayesian computation

by Jonathan U. Harrison and Ruth E. Baker

2020

Main comments

This paper discusses the problem of estimating models with intractable likelihood in an ABC-SMC framework in which there is uncertainty on how to use summary statistics. The methods aim at estimating the optimal weights of summary statistics in order to achieve consistent estimation of the posterior distribution. The proposed method, based on nearest neighbour distance, to find optimal weights is substantially compared against uniform weights and rescaling the weights proposed in Prangle et al.

While I find the approach interesting as based on information theory approach, that is weights are choosen in order to maximize the information given by the used set of summary statistics, I have two major concerns:

1. The methods does not apply when priors are improper priors. Although this is not usual in ABC, in the end we don't know what happen if priors are very spread out on the support of parameter space. For instance, would this imply uniform weights?
2. All comparison with the methods I think are misleading, in fact the obtained posteriors are compared among them but not with the true posterior in the case when this is known by using analytical likelihood. For example, in the case of Uniform or Normal example I would have drawn the true posterior instead of the true value of the parameter which is just a point.
3. Comparison should be done also in terms of computational effort at least in term of the number of simulated data from the model. For instance, the uniform assignment of weights maybe is not optimal, but it is certainly less computational demanding.

Minors comments

- pag 3 lines 72-75 these could be rephrased.
